# Morphologic study of the effect of iron on pseudocyst formation in *Trichomonas vaginalis* and its interaction with human epithelial cells

**DOI:** 10.1590/0074-02760170032

**Published:** 2017-10

**Authors:** Geovane Dias-Lopes, Leonardo Saboia-Vahia, Eliane Trindade Margotti, Nilma de Souza Fernandes, Cássia Luana de Faria Castro, Francisco Odencio Oliveira, Juliana Figueiredo Peixoto, Constança Britto, Fernando Costa e Silva, Patricia Cuervo, José Batista de Jesus

**Affiliations:** 1 Fundação Oswaldo Cruz-Fiocruz, Instituto Oswaldo Cruz, Laboratório de Biologia Molecular e Doenças Endêmicas, Rio de Janeiro, RJ, Brasil; 2 Fundação Oswaldo Cruz-Fiocruz, Instituto Oswaldo Cruz, Laboratório de Pesquisa em Leishmanioses, Rio de Janeiro, RJ, Brasil; 3 Universidade Federal de São João Del Rei, Faculdade de Medicina, Departamento de Medicina, São João Del Rei, MG, Brasil; 4 Instituto de Ensino Superior Presidente Tancredo de Almeida Neves, São João Del Rei, MG, Brasil; 5 Fundação Oswaldo Cruz-Fiocruz, Instituto Oswaldo Cruz, Laboratório de Ultraestrutura Celular, Rio de Janeiro, RJ, Brasil; 6 Universidade Estadual do Norte Fluminense Darcy Ribeiro, Centro de Biociências e Biotecnologia, Laboratório de Biologia Celular e Tecidual, Rio de Janeiro, RJ, Brasil

**Keywords:** Trichomonas vaginalis, iron, pseudocyst, morphological transformation, trophozoites, human epithelial cells

## Abstract

**BACKGROUND:**

*Trichomonas vaginalis* is the aetiological agent of human trichomoniasis, which is one of the most prevalent sexually transmitted diseases in humans. Iron is an important element for the survival of this parasite and the colonisation of the host urogenital tract.

**OBJECTIVES:**

In this study, we investigated the effects of iron on parasite proliferation in the dynamics of pseudocyst formation and morphologically characterised iron depletion-induced pseudocysts.

**METHODS:**

We performed structural and ultrastructural analyses using light microscopy, scanning electron microscopy and transmission electron microscopy.

**FINDINGS:**

It was observed that iron depletion (i) interrupts the proliferation of *T. vaginalis*, (ii) induces morphological changes in typical multiplicative trophozoites to spherical non-proliferative, non-motile pseudocysts, and (iii) induces the arrest of cell division at different stages of the cell cycle; (iv) iron is the fundamental element for the maintenance of typical trophozoite morphology; (v) pseudocysts induced by iron depletion are viable and reversible forms; and, finally, (vi) we demonstrated that pseudocysts induced by iron depletion are able to interact with human epithelial cells maintaining their spherical forms.

**MAIN CONCLUSIONS:**

Together, these data suggest that pseudocysts could be induced as a response to iron nutritional stress and could have a potential role in the transmission and infection of *T. vaginalis*.

*Trichomonas vaginalis* is the aetiological agent of human trichomoniasis, which is one of the most prevalent sexually transmitted infections in humans. Trichomoniasis affects over 270 million people worldwide, and 90% of these infections occur among people living in resource-limited settings (WHO 2012). Although *T. vaginalis* infection is a commonly curable disease and treatment is available at an affordable cost, the emergence of drug resistance and the lack of alternative treatments are increasing concerns among public health policy makers (Meites et al. 2015).

Infection with *T. vaginalis* is associated with increased susceptibility to human immunodeficiency virus, herpes virus and papillomavirus infection, cervical cancer and prostate cancer. Particularly in women, infection of the urogenital tract may lead to pre-term delivery, low-weight births and infertility (Kissinger 2015, Meites et al. 2015).

During their life cycle, trichomonads present a trophozoite form without a true cystic stage. However, the presence of spherical forms with internalised flagella, denominated pseudocysts or endoflagellar forms have been commonly found in these parasites (Lipman et al. 1999, Granger et al. 2000, Pereira-Neves et al. 2003, 2011, 2012, da Costa & Benchimol 2004, Ribeiro et al. 2015, Castro et al. 2016). Pseudocysts are observed mainly under unfavourable growth conditions and, at a low proportion, under *in vitro* axenic culture conditions (Pereira-Neves et al. 2003). In *Tritrichomonas foetus*, a trichomonad that infects the urogenital tract of cattle, pseudocysts induced by iron depletion are reversible and viable (Castro et al. 2016). In *T. vaginalis*, pseudocysts have been observed naturally in patients with cervical neoplasia (Afzan & Suresh 2012) or induced *in vitro* by iron depletion (de Jesus et al. 2007).

In parasitic trichomonads, iron participates in the following: (i) sustaining the parasite’s growth (Lehker & Alderete 1992), (ii) modulating virulence (Kummer et al. 2008, Cárdenas-Guerra et al. 2013); (iii) hydrogenosomal metabolism via essential iron-sulfur [Fe-S] proteins (Vanáčová et al. 2001); (iv) regulating the expression of adhesins and cysteine peptidases involved in cytoadherence and cytotoxicity (Álvarez-Sánchez et al. 2007, Meza-Cervantez et al. 2011); (v) haemolysis (Cárdenas-Guerra et al. 2013); (vi) modulating the expression of ecto-ATPases and ecto-phosphatases (de Jesus et al. 2006); (vii) regulating Myb-like transcription factors (Hsu et al. 2012); (viii) inducing apoptosis in human epithelial cells (Kummer et al. 2008); and (ix) down-regulating the surface immunogen P270 (Alderete et al. 1999).

In previous studies, we observed that iron regulates protein expression in *T. vaginalis* and could be involved in the maintenance of the trophozoite morphology of this species (de Jesus et al. 2007). In the present study, we conducted a comprehensive morphologic analysis of the effect of iron on *T. vaginalis*. We used an approach that employs the removal of this metal from the culture medium by adding the iron chelator 2,2-dipyridyl. Thus, we investigated the effects of iron on parasite proliferation in the dynamics of pseudocyst formation and morphologically characterised the iron depletion-induced pseudocysts using structural and ultrastructural analyses by light microscopy, scanning electron microscopy and transmission electron microscopy. Furthermore, we evaluated the ability of the pseudocyst to interact with host cells and discussed the role of these forms in the life cycle of *T. vaginalis* and its relevance for the transmission and diagnosis of trichomoniasis.

## MATERIALS AND METHODS

*Chemicals* - All the reagents were purchased from Sigma (St. Louis, MO, USA) or Merck (São Paulo, SP, Brazil). Milli-Q-purified water (Millipore Corp., Bedford, MA, USA) was used for all solutions. The iron chelator 2,2-dipyridyl is an organic, synthetic, membrane-permeable compound that associates with extracellular and intracellular iron (Breuer et al. 1995).

*Microorganisms and culture procedures* - The FMV1 strain of *T. vaginalis* (de Jesus et al. 2004) was cultivated at 37ºC in trypticase yeast extract maltose medium (TYM), pH 6.3, supplemented with 10% heat-inactivated bovine serum and 0.6 mM FeSO_4_ (standard TYM medium). For pseudocyst induction, parasites cultivated in standard TYM medium were collected at the logarithmic phase of growth by centrifugation at 1400 × *g* for 5 min at 4ºC, washed twice with PBS, pH 7.2, and incubated at 37ºC for 48 h in TYM medium, pH 6.3, supplemented with 10% heat-inactivated bovine serum and 180 µM 2,2-dipyridyl (TYM-DIP inducer medium). The parasite densities and/or morphotypes were estimated by counts in a haemocytometer. In all assays, parasite viability was estimated using the Trypan blue dye exclusion test (0.4% in sterile PBS). Parasite cultures with viability above 90% were used.

*Growth curve and effect of iron chelator on parasite proliferation* - To evaluate the influence of iron chelation on parasite proliferation, 1×10^5^/mL parasites were inoculated in 10 mL of standard TYM medium or TYM-DIP inducer medium and were incubated at 37ºC for 72 h. The cellular density was evaluated daily by counting in a haemocytometer. Three independent assays were carried out in triplicate.

*Parasite morphotypes and light microscopy analysis* - The parasites were cultivated for 48 h at 37ºC in standard TYM medium or TYM-DIP inducer medium. The counting of parasites morphotypes was performed every 12 h over 48 h in triplicate using a haemocytometer. For micrograph recording, the parasites cultivated in standard TYM medium or TYM-DIP inducer medium were harvested by centrifugation at 1400 × *g* for 5 min at 4ºC, washed with PBS, pH 7.2, or PBS pH 7.2 containing 180 µM 2,2-dipyridyl (PBS-DIP), respectively, and fixed in 2.5% glutaraldehyde. The parasite morphotypes were observed and counted by differential interference contrast microscopy (DIC) or on slides stained with the Panotico LB kit®(Laborclin, Brazil). Spherical, non-proliferative, non-motile parasites with all flagella internalised were considered as typical pseudocysts.

*Analysis of the morphological reversibility of iron depletion-induced pseudocysts* - To evaluate whether iron depletion-induced pseudocysts are viable and reversible forms, the parasites cultivated for 48 h in TYM-DIP inducer medium were washed twice with PBS-DIP, inoculated in fresh standard TYM medium and incubated at 37ºC for 24 h. Parasite morphology was examined by DIC, and the morphotypes were counted in a haemocytometer. Three independent assays were carried out in triplicate.

*Scanning electron microscopy analysis* - The parasites (1×10^5^/mL cells) were inoculated in standard TYM medium or TYM-DIP inducer medium for 48 h at 37ºC. The parasites were then collected, washed and concentrated as described above, and a total of 5x10^6^ parasites were fixed with 2.5% glutaraldehyde in 0.1 M Na-cacodylate buffer (pH 7.2) at room temperature for 1 h at 25ºC and post-fixed with a solution of 1% OsO_4_, 0.8% potassium ferricyanide and 2.5 mM CaCl_2_ in the same buffer for 1 h at 25ºC. The samples were dehydrated in an ascending acetone series and dried by the critical point method with CO_2_, mounted on aluminium stubs, coated with a 20-nm-thick gold layer and were examined with a Jeol JSM6390LV scanning electron microscope (Tokyo, Japan).

*Transmission electron microscopy analysis* - The parasites (1×10^5^/mL cells) were inoculated in standard TYM medium or TYM-DIP inducer medium for 48 h at 37ºC. Thereafter, the parasites were collected, washed and concentrated as described above, and a total of 5x10^6^ parasites were fixed with 2.5% glutaraldehyde in 0.1 M Na-cacodylate buffer (pH 7.2) at room temperature for 40 min at 25ºC and post-fixed with a solution of 1% OsO_4_, 0.8% potassium ferricyanide and 2.5 mM CaCl_2_ in the same buffer for 20 min at 25ºC. The parasites were then dehydrated in an ascending acetone series and were embedded in PolyBed 812 resin. Ultrathin sections were stained with uranyl acetate and lead citrate and were examined in a Jeol JEM1011 transmission electron microscope (Tokyo, Japan).

*Effect of FeSO*_*4*_ *on iron depletion-induced pseudocysts* - To demonstrate that the maintenance of the typical pear-shaped trophozoite morphology of *T. vaginalis* is dependent on iron, the parasites were cultivated for 48 h in TYM-DIP inducer medium, and then, 1.2 mM FeSO_4_ was added and incubated for an additional 24 h. Next, the morphotypes were determined by counting with a haemocytometer. Three independent assays were carried out in triplicate.

*Analysis of T. vaginalis-HeLa cell interactions by scanning electron microscopy* - HeLa cells (ATCC CCL-2.1 HeLa 229) were cultured in 24-well plates in RPMI medium supplemented with 10% bovine serum and maintained at 37ºC in an atmosphere of 5% CO_2_ until confluence. Parasites cultivated in standard TYM medium or TYM-DIP inducer medium for 48 h at 37ºC were submitted to interactions with a HeLa cell monolayer (5:1 parasites:HeLa cells) in RPMI medium supplemented with 10% heat-inactivated bovine serum and containing or not containing 180 µM 2,2-dipyridyl. The interaction was allowed to proceed for 30 min at 37ºC and 5% CO_2_. To quantify adhered parasites, after 30 min, the interaction assays were analysed using inverted phase-contrast microscopy with a 40X objective lens, and the percentage of adhered and non-adhered parasites was determined from counts of 400 parasites per well. Additionally, samples were fixed on slides and stained with the Panotico LB kit® to observe the morphology by light microscopy, or parasites were fixed and prepared for scanning electron microscopy analysis as described above.

*Analysis of T. vaginalis-VEC interactions by light microscopy* - Human vaginal epithelial cells (VECs) obtained from a young healthy donor were extensively washed with PBS pH 7.2 and resuspended in RPMI at 37ºC. Parasites (1×10^5^ cells) cultivated in 10 mL of standard TYM medium or TYM-DIP inducer medium were collected by centrifugation, washed in PBS or PBS-DIP, respectively, and then added to the VEC suspension in 500 μL RPMI medium supplemented with 10% heat-inactivated bovine serum and containing or not containing 180 µM 2,2-dipyridyl. The cell-parasite interaction was allowed to proceed for up 30 min. To quantify adhered parasites, after the interaction, the cell suspension was homogenised and 50 μL was distributed in microscopy slides (10 in total) and covered with coverslips, and the number of adhered parasites was estimated by counting in 20 random fields in each slide using DIC microscopy. In addition, samples were fixed on slides and stained with the Panotico LB kit® to observe the morphology of adhered parasites using light microscopy.

## RESULTS

**Fig. 1:**
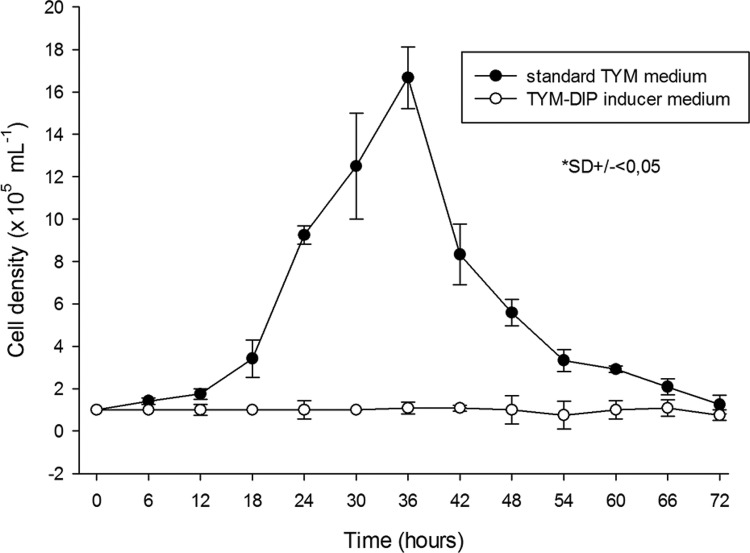
: effect of iron depletion on the cell growth of the *Trichomonas vaginalis* FMV-1 strain. Parasites were cultivated in standard TYM medium (filled circle) or TYM-DIP inducer medium (empty circle) for 72 h at 37ºC. The parasites were counted every 6 h using a haemocytometer. The experiments were performed in triplicate, and the results are shown as medians ± SD.

*Iron depletion inhibits T. vaginalis proliferation* - Comparison of the growth curves of *T. vaginalis* cultivated in standard TYM medium or TYM-DIP inducer medium for 72 h at 37ºC showed that iron depletion inhibited parasite proliferation (Fig. 1). The parasites cultivated in standard TYM medium reached a log phase peak at 36 h with a maximum cell density of 1.7 × 10^6^ cells/mL, followed by a strong decline in cell density (Fig. 1). In contrast, parasites cultivated in TYM-DIP inducer medium did not show significant growth but remained alive for up to 72 h.

**Fig. 2:**
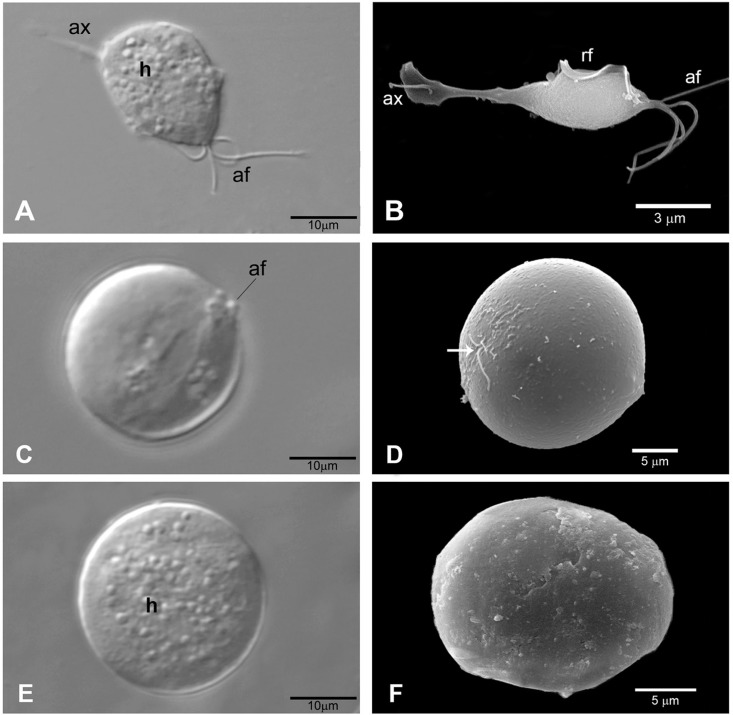
: effect of iron depletion on the morphology of *Trichomonas vaginalis*. Differential interference contrast microscopy (DIC) micrographs (A, C, E) and scanning electron microscopy (B, D, F) of *T. vaginalis*. Parasites cultivated in standard TYM medium for 48 h (A-B) show typical pear-shaped trophozoites with all flagella visible. Parasites cultivated in TYM-DIP inducer medium for 48 h (C-F) exhibited spherical forms at different stages of flagella internalisation (C, D), and typical pseudocysts with all flagella internalised (E, F). The arrow in D indicates the localisation of flagellar internalisation. ax: axostyle; af: anterior flagella; n: nucleus; h: hydrogenosomes.

*Light microscopy and ultrastructural analysis of the effect of iron depletion on the morphology of T. vaginalis* - Differential interference contrast microscopy (DIC) micrographs of parasites cultivated in standard TYM medium for 48 h showed typical pear-shaped trophozoite forms with four anterior flagella and a recurrent flagellum, an axostyle and an eccentric nucleus (Fig. 2A). Hydrogenosomes were clearly observed along the cell body (Fig. 2A, E). The parasites cultivated for 48 h in TYM-DIP inducer medium exhibited typical spherical forms of different diameters, with flagella at the different stages of internalisation, slightly motile or non-motile (as observed previous to fixation), without a visible external axostyle (Fig. 2C). The process of flagellar internalisation of some cells seems to involve the internalisation of the recurrent flagellum first, followed by the anterior flagella. Typical spherical and non-motile pseudocysts with all internalised flagella were clearly observed (Fig. 2E).

**Fig. 3:**
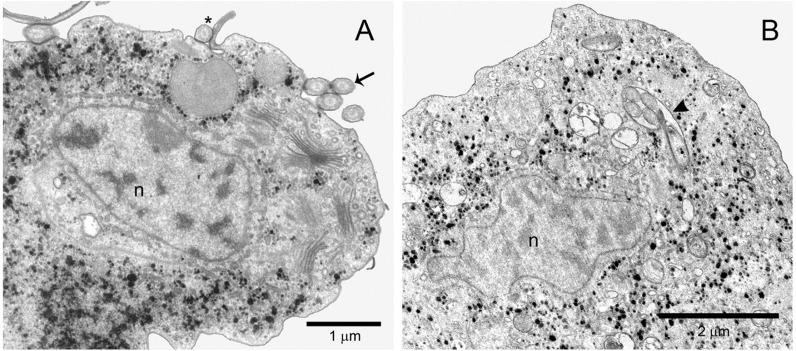
: transmission electron microscopy (TEM) of longitudinal thin sections of *Trichomonas vaginalis*. Parasites cultivated in either standard TYM medium for 48 h (A) or in TYM-DIP inducer medium for 48 h (B). (A) Typical trophozoite exhibiting externalised recurrent flagellum (*) and four anterior flagella structures (arrow). (B) Pseudocysts phenotype exhibit internalised flagella (arrow head). n: nucleus.

Scanning electron microscopy of parasites cultivated in standard TYM medium for 48 h revealed typical trophozoites commonly found in axenic culture, exhibiting all anterior flagella and a recurrent flagellum associated with an undulant membrane (Fig. 2B). In contrast, parasites cultivated in TYM-DIP inducer medium for 48 h underwent a drastic morphological transformation from typical trophozoites to pseudocysts, which exhibit spherical forms, internalising anterior flagella and a recurrent flagellum (Fig. 2D, F). Some pseudocysts at different stages of flagella internalisation were also observed (Fig. 2D). Such observations were further corroborated by TEM analysis (Fig. 3). TEM clearly showed that parasites cultivated in TYM standard medium exhibited externalised flagella (Fig. 3A), whereas parasites cultivated in TYM-DIP inducer medium exhibited all internalised flagella (Fig. 3B).

**Fig. 4:**
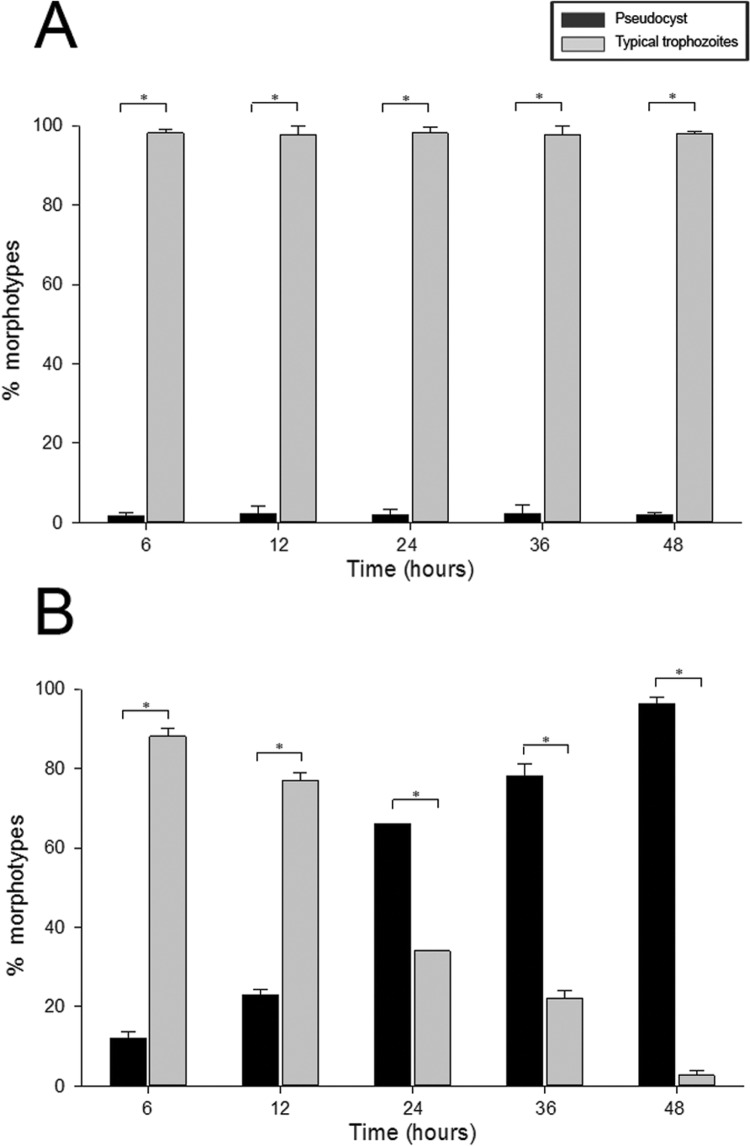
: effect of iron depletion on parasite phenotypes. Parasites were cultivated for 48 h in standard TYM medium (A) or TYM-DIP inducer medium (B). Counting of the parasite phenotypes was performed in triplicate using a haemocytometer. The results are presented as the means and standard errors from three independent experiments. *Significant differences between morphotypes (p < 0.001).

*Effect of iron depletion on the morphology of T. vaginalis* - The examination of the morphology of the *T. vaginalis* population cultivated in TYM standard medium or TYM-DIP inducer medium showed that iron depletion induced an increased percentage of pseudocysts over time compared with parasites cultivated in iron-rich medium (Fig. 4). In the control medium, approximately 95% of parasites presented the typical pear-shape motile trophozoite form during the experimental course (Fig. 4A). Remarkably, under this control condition, the parasite population contained nearly 5% pseudocysts (Fig. 4A). In contrast, in the TYM-DIP inducer medium, an increasing percentage of pseudocysts was observed over time (Fig. 4B). As early as 6 h of culture, it was possible to observe ~10% pseudocysts, and this number increased steadily over time, reaching 97% pseudocyst forms at 48 h (Fig. 4B).

**Fig. 5:**
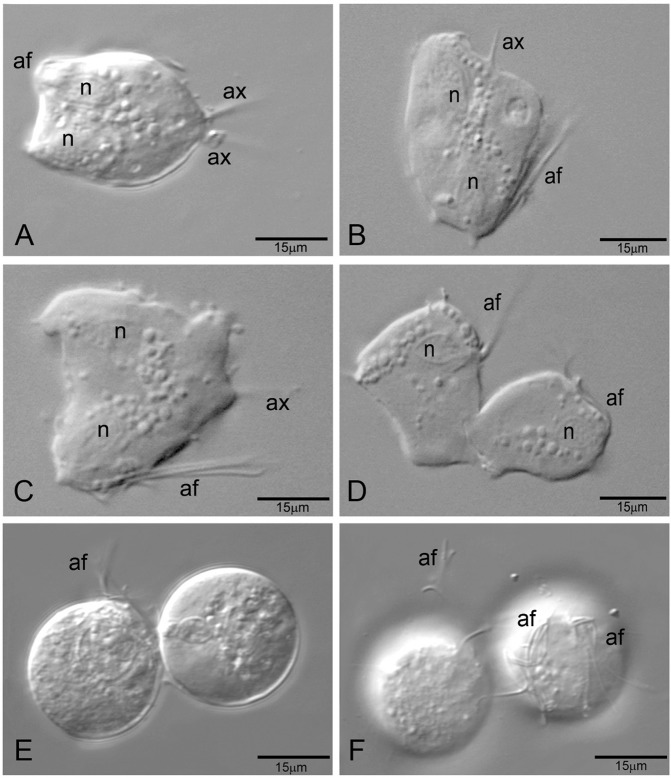
: effect of iron depletion on the division process of *Trichomonas vaginalis*. Differential interference contrast microscopy (DIC) of *T. vaginalis* cultivated in standard TYM medium for 48 h (A-D) showed that trophozoites underwent the cell division process; parasites cultivated in TYM-DIP inducer medium for 48 h (E, F) showed parasites with arrested cell division. ax: axostyle; af: anterior flagella; n: nucleus.

*Effect of iron depletion on the division process of T. vaginalis* - The parasite population cultivated in standard TYM medium for 48 h exhibited cells in different phases of mitosis (Fig. 5A-D). In contrast, the cultivation of parasites in TYM-DIP inducer medium revealed parasites at different stages of flagella internalisation, showing a single nucleus or two nuclei and duplicated anterior flagella, indicating an interruption of the cell division process in different stages of the cell cycle (Fig. 5E, F).

**Fig. 6:**
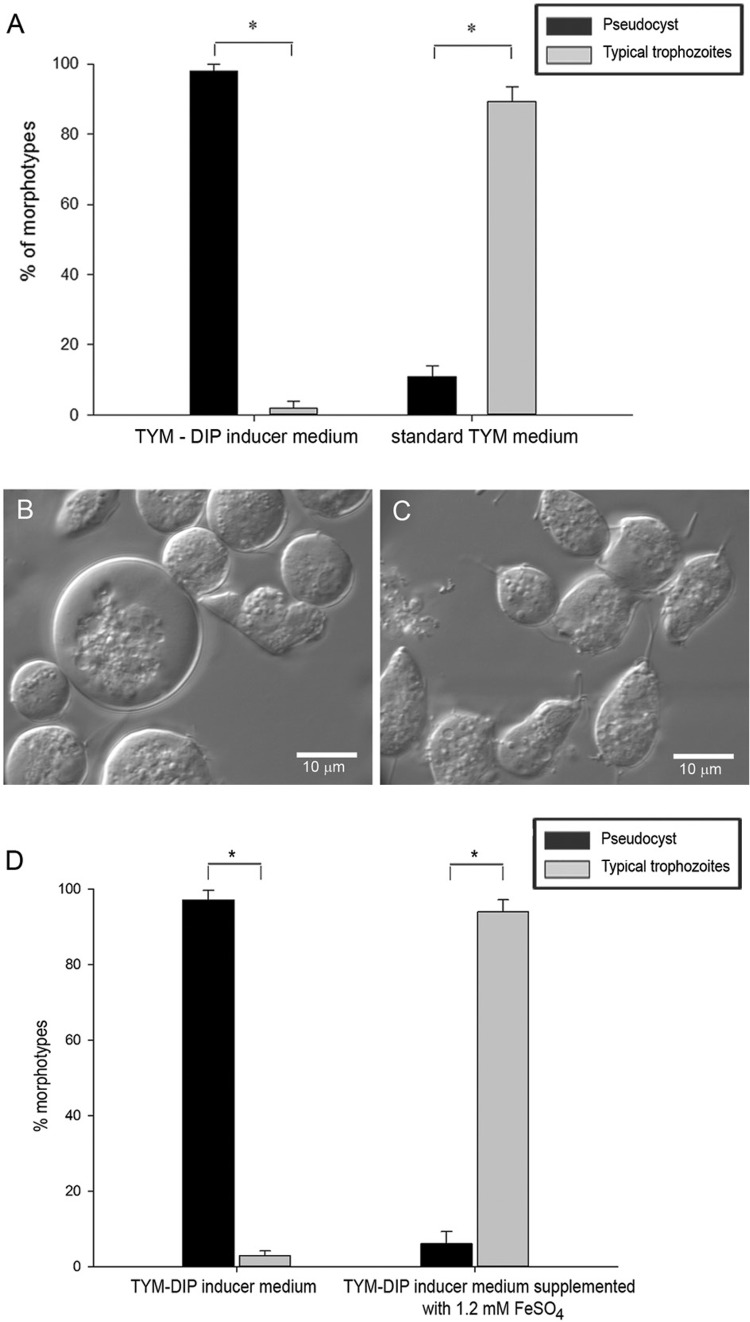
: phenotype reversibility of iron depletion-induced pseudocysts. (A) Parasites maintained for 48 h in TYM-DIP inducer medium (left) were collected, washed in PBS, and subsequently inoculated in fresh standard TYM medium (right) and incubated for 24 h. (B) DIC of *Trichomonas vaginalis* cultivated in TYM-DIP inducer medium for 48 h. (C) DIC of *T. vaginalis* maintained for 48 h in TYM-DIP inducer medium and subsequently inoculated in fresh standard TYM medium and incubated for 24 h. The micrographs show pseudocysts of different sizes (B) or typical pear-shaped trophozoites (C). (D) Effect of FeSO4 on the morphology of iron depletion-induced pseudocysts. Morphotypes of *T. vaginalis* cultivated in TYM-DIP inducer medium for 48 h (left), or TYM-DIP inducer medium for 48 h and then were supplemented with 1.2 mM FeSO4 and cultivated for an additional 24 h (right) were estimated by counting in a haemocytometer. The data obtained from three independent experiments are presented as the means ± standard error. *Significant differences between morphotypes (p < 0.001).

*Phenotype reversibility assay and the effect of FeSO*_*4*_ *on the morphology of iron depletion-induced pseudocysts* - To analyse whether iron depletion-induced pseudocysts are viable cellular forms and whether such a phenotype is reversible to a typical trophozoite one, parasites cultivated for 48 h in TYM-DIP inducer medium were reinoculated into standard TYM medium and incubated for additional 24 h. In this assay, a population of *T. vaginalis* cultivated for 48 h in TYM-DIP inducer medium and exhibiting approximately 97% pseudocysts (Fig. 6A, B) was inoculated into standard TYM medium; after 24 h of incubation, the morphotypes observed were mostly (~90%) typical trophozoites (Fig. 6A, C). The remaining 10% of the parasite population was composed of pseudocyst and parasite forms in the intermediate transformation process.

To evaluate whether iron supplementation could recover the trophozoite morphology and proliferation capability of iron-depletion-induced *T. vaginalis* pseudocysts, parasites cultivated for 48 h in TYM-DIP inducer medium were supplemented with 1.2 mM FeSO_4_ and were cultivated for an additional 24 h. Morphotype evaluation showed that iron supplementation indeed recovered typical trophozoite morphology for ~93% of the parasite population, whereas ~7% maintained the pseudocyst form (Fig. 6D). This percentage of pseudocysts was similar to that of the pseudocyst population observed in the control (parasites cultivated in standard TYM medium).

**Fig. 7:**
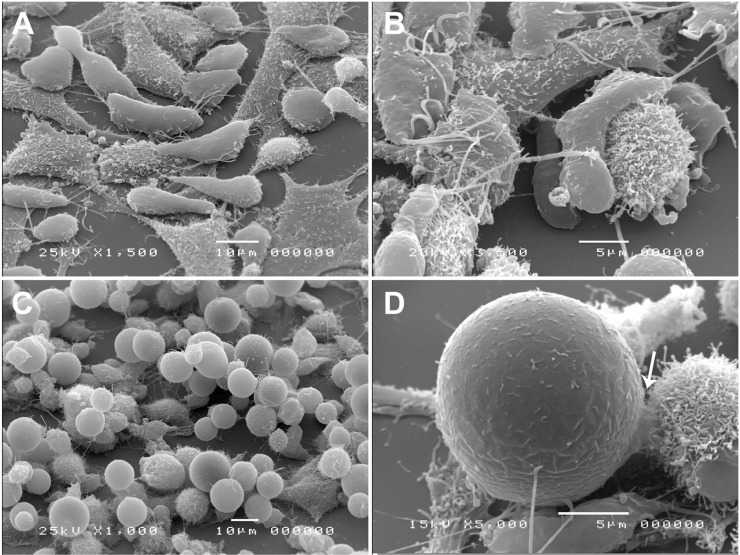
: *Trichomonas vaginalis*-HeLa cell interactions. Representative scanning electron microscopy (SEM) images of the morphology of trophozoites (A-B) or iron-depletion induced pseudocysts (C-D) during interaction with the HeLa cell monolayer. (C) A cluster of pseudocysts was observed. The arrow indicates membrane projections during contact with the host cell membrane.

*Interaction of T. vaginalis with HeLa and vaginal epithelial cells (VECs)* - Interaction of trophozoites or iron-depletion induced pseudocysts with HeLa cells was evaluated by SEM. Trophozoites exhibited high plasticity, yielding an amoeboid morphology after adhesion to the epithelial cells (Fig. 7A, B). Remarkably, pseudocysts were also able to adhere to HeLa cells, maintaining their spherical forms and emitting discreet membrane projections during contact with the host cell membrane (Fig. 7C, D, arrow).

**Fig. 8:**
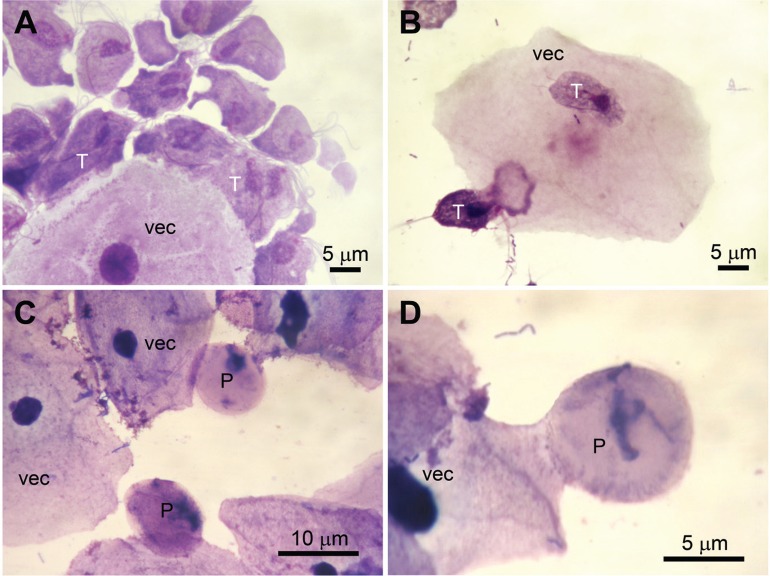
: interaction of *Trichomonas vaginalis* with VECs. Light microscopy of trophozoites-VEC interactions (A, B) or iron-depletion induced pseudocyst-VEC interactions (C, D). Samples were stained with Panotico LB kit®. T: trophozoite; P: pseudocyst.

Similar to that observed in the interaction with HeLa cells, trophozoites readily adhered to VECs, changing their typical ellipsoid forms to adherent amoeboid trophozoites (Fig. 8A, B). Amoeboid parasites exhibited multiple cytoplasmic projections, which mediated the parasite-VEC contact. In contrast, pseudocysts conserved their spherical forms during interaction with VECs but were able to adhere to the cells (Fig. 8C, D).

**Fig. 9:**
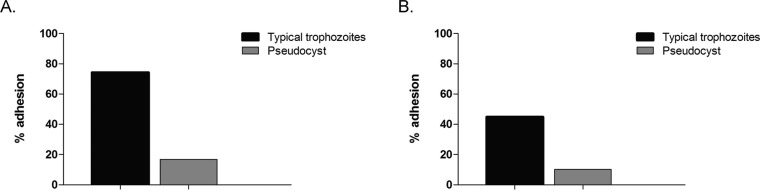
: percentage of adhesion of *Trichomonas vaginalis* trophozoites and iron-depletion induced pseudocysts to epithelial cells. The figure shows the rate of adhesion of trophozoites or iron-depletion induced pseudocysts to HeLa cells (A) or VECs (B).

The percentages of adhesion of trophozoites and pseudocysts to HeLa cells were 74% and 16%, respectively (Fig. 9A), whereas for VECs, the percentages of adhesion were 44.9% and 10,2% for trophozoites and pseudocysts, respectively (Fig. 9B).

## DISCUSSION

In this study, we investigated the morphological alterations of *T. vaginalis* induced by iron depletion and verified the role of iron during the interaction of the parasite with epithelial cells. The results obtained here show that iron depletion (i) interrupts the proliferation of *T. vaginalis*; (ii) induces morphological changes in typical multiplicative trophozoites to spherical non-proliferative, non-motile pseudocysts; and (iii) induces the arrest of cell division at different stages of the cell cycle. Remarkably, we observed that (iv) iron is the fundamental element for the maintenance of typical trophozoite morphology; (v) pseudocysts induced by iron depletion are viable and reversible forms; and finally, we demonstrated that (vi) pseudocysts induced by iron depletion are able to interact with human epithelial cells, maintaining their spherical forms.

Here, we further analysed the effect of iron depletion on the morphological changes of trophozoites using structural and ultrastructural approaches. We observed that the inoculation of the trophozoite population from an iron-rich standard medium into an iron-depleted medium immediately interrupts parasite proliferation and induces concomitant morphological transformation to pseudocysts, which are parasite forms without apparent motility. Such a result shows that pseudocysts are non-replicative forms. Accordingly, the previous results of our group showed that iron depletion from *T. vaginalis* culture medium leads to a significant reduction in the expression of crucial proteins involved in the regulation of the cell cycle, protein synthesis, proteolysis and hydrogenosomal metabolism (de Jesus et al. 2007). Drastic alterations in protein synthesis, low metabolic activity and pseudocysts being non-motile parasites support the idea that pseudocysts are the non-replicative form of *T. vaginalis* under iron-depletion conditions. Although several studies have shown the deleterious effect of iron depletion on *T. vaginalis* proliferation and other parasites (Lehker & Alderete 1992, Melo-Braga et al. 2003, d, de Jesus et al. 2006, 20, 2007, Mesquita-Rodrigues et al. 2013 , Hernández-Cuevas et al. 2014 , Olmo et al. 2014; Thipubon et al. 2015 , Castro et al. 2016), the association of iron depletion with morphological alteration from trophozoites to pseudocysts has not been assessed. Thus, we demonstrated that, in addition to its role in the regulation of parasite proliferation, iron also regulates parasite morphology. Because 2,2-dipyridyl is a lipophilic chelator that chelates iron from both the extracellular and intracellular milieu, the interruption of parasite proliferation could be due to (i) the interruption of the transcription of genes whose promoters are dependent on iron and/or (ii) the decreased or disrupted synthesis of essential [Fe-S] proteins that are crucial for the metabolism of parasites (Vanáčová et al. 2001, Hsu et al. 2012, Beltrán et al. 2013).

We observed that pseudocysts exhibit up to two nuclei. We believe that such an observation is not due to pseudocyst division but to the interruption of the cell division of parasites when they were inoculated into the TYM-DIP inducer medium - i.e., when log-phase trophozoites undergoing division in the pre-inoculum standard TYM medium were inoculated in TYM-DIP inducer medium, they suffered arrest of the cell cycle and concomitantly suffered transformation to pseudocysts. We corroborated that pseudocysts are non-proliferative forms by the lack of an increased cellular density during the growth curve assay in TYM-DIP inducer medium. Bi- and multinucleated pseudocysts have also been observed in *T. vaginalis* (Abonyi 1995) and *T. foetus* (Pereira-Neves & Benchimol 2009), including in fresh preputial smegma from infected bulls (Pereira-Neves et al. 2011).

Trophozoites undergo a transformation from ellipsoid or amoeboid forms to rounded cells in which the flagella are internalised and axostyles are retracted. The formation of pseudocysts occurs in other trichomonads, including *T. tenax* (Ribeiro et al. 2015) and *T. foetus*, when subjected to distinct physical or chemical stimuli in the medium (Granger et al. 2000, Pereira-Neves et al. 2003, da Costa & Benchimol 2004). We previously observed that the alteration of parasite morphology due to iron depletion was followed by a significantly different expression of cytoskeleton-related proteins and proteins involved in cell signalling (de Jesus et al. 2007). The cell surface of trichomonads exhibits an active endocytic machinery (Benchimol et al. 1986, 19, 1990) and it has been suggested that during pseudocyst formation, the flagella are internalised by a receptor-mediated endocytosis process (Granger et al. 2000, Pereira-Neves et al. 2003). In addition, we observed that the sequence of flagellar internalisation was similar to that observed in *T. foetus*, where the recurrent flagellum is internalised first, followed by the anterior flagella (Pereira-Neves et al. 2003). Accordingly, the expression of several proteins involved in membrane remodelling and phagocytosis were modulated by iron depletion in *T. vaginalis* (de Jesus et al. 2007).

We observed that pseudocysts from TYM-DIP inducer medium were able to recover their trophozoite form and resume proliferation after re-inoculation in fresh standard TYM medium. Such results demonstrated that pseudocysts induced by iron depletion are viable and reversible forms. Accordingly, other authors have shown that pseudocysts of trichomonads represent a reversible form of the parasite that may occur as a response to drastic changes in the environment (Lipman et al. 1999, Pereira-Neves & Benchimol 2009). We also observed that iron is the key regulator of trophozoite morphology because the addition of FeSO_4_ to TYM-DIP inducer medium - i.e., chelator-containing medium - reverted pseudocysts to trophozoites and recovered the proliferation capability of the parasite. Additionally, it has been shown that *T. vaginalis* pseudocysts occur naturally in the host, suggesting that these forms may coexist with replicative trophozoites (de Jesus et al. 2004, Afzan & Suresh 2012). The natural occurrence of *T. foetus* pseudocysts was also observed in fresh bull preputial secretion, exhibiting a higher proportion of pseudocysts than that observed in culture *in vitro* (Pereira-Neves et al. 2011).

We also evaluated the capability of the pseudocysts to interact with human epithelial cells. In fact, we observed that although the adhesion percentage of pseudocysts was lower than that for trophozoites, pseudocysts induced by iron depletion were able to interact and adhere to HeLa cells and VECs. Such results, together with the previous observations that pseudocysts are found naturally in the host (de Jesus et al. 2004, Afzan & Suresh 2012), suggest that pseudocysts could persist in the vaginal environment by adhering to host epithelial cells to avoid being carried out by menstrual fluid, for example. Alternatively, during the parasite life cycle, pseudocysts could be released during vaginal discharge, which could favour their dispersion and successful infection. Therefore, knowledge about the biology of pseudocyst formation is important not only to better understand the life cycle of *T. vaginalis* but also to understand their role in the dynamics of parasite transmission. Currently, in many countries, including Brazil, the diagnosis of trichomoniasis is based only on the detection of motile trophozoites in fresh clinical specimens; therefore, false-negative diagnoses may occur during the laboratory analysis of clinical specimens. Searching for pseudocyst forms should also be considered during the diagnosis of trichomoniasis by wet-mount microscopic examination or by culturing methods.
